# Voltage and Power-Controlled Regimes in the Progressive Unipolar RESET Transition of HfO_2_-Based RRAM

**DOI:** 10.1038/srep02929

**Published:** 2013-10-14

**Authors:** Shibing Long, Luca Perniola, Carlo Cagli, Julien Buckley, Xiaojuan Lian, Enrique Miranda, Feng Pan, Ming Liu, Jordi Suñé

**Affiliations:** 1 Lab of Nanofabrication and Novel Device Integration, Institute of Microelectronics, Chinese Academy of Sciences, Beijing 100029, People's Republic of China; 2 Departament d'Enginyeria Electrònica, Universitat Autònoma de Barcelona, Bellaterra 08193, Spain; 3 CEA, LETI, MINATEC Campus, 17 rue des Martyrs, F-38054 Grenoble Cedex 9, France; 4 Laboratory of Advanced Materials, Department of Materials Science and Engineering, Tsinghua University, Beijing 100084, People's Republic of China

## Abstract

Resistive switching (RS) based on the formation and rupture of conductive filament (CF) is promising in novel memory and logic device applications. Understanding the physics of RS and the nature of CF is of utmost importance to control the performance, variability and reliability of resistive switching memory (RRAM). Here, the RESET switching of HfO_2_-based RRAM was statistically investigated in terms of the CF conductance evolution. The RESET usually combines an abrupt conductance drop with a progressive phase ending with the complete CF rupture. RESET1 and RESET2 events, corresponding to the initial and final phase of RESET, are found to be controlled by the voltage and power in the CF, respectively. A Monte Carlo simulator based on the thermal dissolution model of unipolar RESET reproduces all of the experimental observations. The results contribute to an improved physics-based understanding on the switching mechanisms and provide additional support to the thermal dissolution model.

The charge-based flash memory, which dominates the huge non-volatile memory (NVM) market, faces serious scaling limitations due to the difficulty of confining electrons within thinner and leakier potential barriers and has some intrinsic disadvantages such as a high operation voltage, a long program/erase time, and a limited endurance. Resistive switching memory (RRAM) is widely accepted to have a great potential to overcome these limitations and thus is a powerful candidate for future NVM and storage class memory because of its simple structure, good scalability, high speed, low power and ease of integration with CMOS back-end-of-the-line processes^1^,^2^,^3^,^4^,^5^,^6^. In RRAM, data storage is achieved through the reversible resistive switching (RS) between at least two stable resistance states, the high resistance state (HRS or OFF-state) and the low resistance state (LRS or ON-state). RRAM devices, which have been identified as a solid-state implementation of a memristive system, are also very promising for reconfigurable logic applications and for neuromorphic computer architectures^7^,^8^,^9^. RS is usually induced with a ramped or pulsed electrical stress of the same (unipolar switching) or opposite polarity (bipolar switching) and has been reported in a wide variety of materials, including binary oxides^10^,^11^,^12^,^13^,^14^,^15^,^16^,^17^ and complex perovskite oxides^18^,^19^, etc., among which HfO_2_ is one of the most widely investigated and most competitive resistive switching functional materials. To successfully push RRAM into applications, it is imperative to solve some key issues, such as revealing the physics underlying the RS phenomena^20^,^21^,^22^,^23^,^24^,^25^,^26^ with special emphasis on the related statistics^27^,^28^,^29^,^30^,^31^ and the effective control of the statistical variation of the switching parameters^32^. For the filament-type RRAM devices, the mechanism of the SET transition from HRS to LRS or the conductive filament (CF) formation is comparatively clear and is interpreted as a defect-induced soft-breakdown, involving the oxidation/reduction (redox) processes of metal cations or oxygen anions in the interfaces and bulk of the RS layer and their migration through this layer^2^,^20^,^31^. However, the mechanism of the RESET transition or the CF rupture is more complicated. The electrochemical reactions and/or Joule heat might play a crucial role^2^,^33^,^34^,^35^,^36^,^37^. Furthermore, the microscopic nature of the CF is still under discussion. On the other hand, the detailed process of the CF rupture and the physics of the RESET transition has not yet been fully addressed.

In this work, we systematically investigate the RESET process of Pt/HfO_2_/Pt RRAM structures operated in the unipolar mode^13^. The switching occurs by the destruction and reformation of a CF due to redox processes involving the generation and movement of oxygen vacancies^13^,^30^,^31^. In a typical RESET cycle with a voltage ramp, an abrupt current jump event (RESET1) usually occurs just after reaching the maximum current, followed by a progressive RESET phase consisting of several successive smaller current drops between well-defined intermediate states with the conductance falling between those of the LRS and the HRS. This progressive RESET phase precedes the complete CF rupture, which is detected as a final abrupt current jump (RESET2) of several orders of magnitude. The statistical analysis of the evolution of the current, the voltage and the power reveals the existence of two different RESET regimes. For the first time we found that RESET1 is determined by the voltage applied to the CF while the progressive RESET stage and RESET2 are determined by the power dissipated in the CF. The transition from the voltage-controlled to the power-controlled regime depends on the CF conductance and the series resistance. To further understand these RESET processes, the evolution of the statistical distribution of conductance is reported as a function of the voltage, and all of the experimental observations are reproduced with a Monte Carlo simulator based on the thermal dissolution model^29^,^30^,^33^,^34^,^35^.

## Results

**Figure 1:**
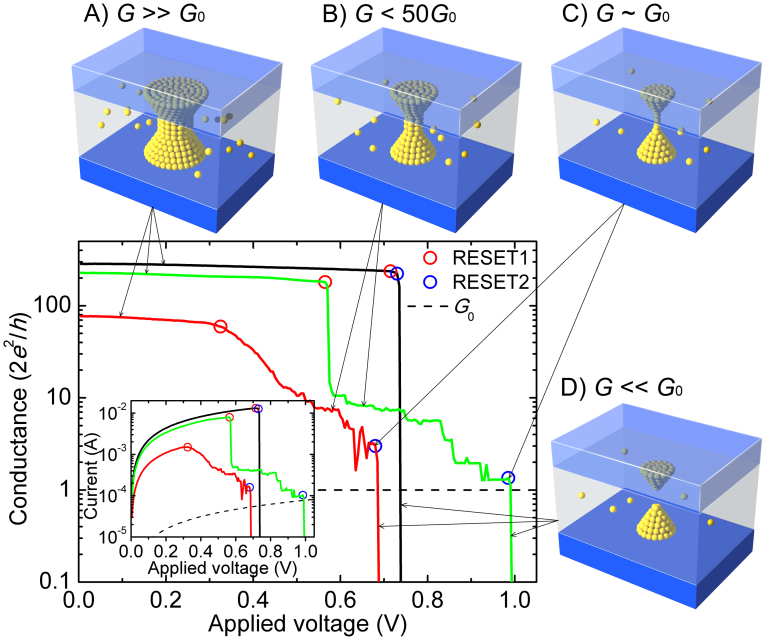
Three typical experimental *G*–*V* and *I*–*V* curves showing abrupt RESET (black curves), RESET in several successive jumps (green curves) and progressive RESET (red curves). Insets A to D show the different stages of the CF during the RESET processes.

The studied Pt/HfO_2_/Pt RRAM device^13^ (see Methods Section) displays unipolar switching. Figure 1 shows some typical RESET current-voltage (*I*–*V*) and conductance-voltage (*G*–*V*) curves in which the conductance, defined as *I*/(*VG*_0_) in units of the quantum of conductance *G*_0_ = 2*e*^2^/*h*, corresponds to the series combination of the CF resistance and the access series resistance, which has been estimated to be *R*_S_ ~ 28 Ω^30^ (see Figure S1 in Supplementary Information). A single channel quantum wire (inset C in Figure 1) with a conductance of ~*G*_0_^38^,^39^, represents a natural boundary between two qualitatively different electron transport regimes. One corresponds to a continuous CF (insets A and B), in the sense that the electron wave functions are extended along the conduction path. The other is related to a broken CF (inset D) in which the conduction is limited by a potential barrier, i.e., a CF with a gap in which the electron transport occurs by hopping or tunneling. Notice that the final current jump (RESET2) corresponding to the CF rupture always crosses the *G*_0_ boundary. In the majority of the cases (green curves in Figure 1), a large and abrupt current drop occurs just after reaching the maximum current (RESET1 marked with red circles), followed by successive current drops of a smaller magnitude between several intermediate states (inset B). These intermediate states reveal the discrete nature of the CF and suggest its evolution through different atomic-size configurations of a reduced cross-section before a gap is fully opened at RESET2 (blue circles). In other switching cycles (red curves), the current reduction is rather progressive, and only the final current jump is evident. In a few cases, the RESET1 leads to the complete CF disconnection such that RESET1 and RESET2 coincide. These abrupt RESET transitions (black curves) are usually observed when the CF has a high initial ON-state conductance (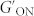) and reaches very high RESET currents. On the other hand, fully progressive RESET transients usually occur when the CF has a low initial conductance.

**Figure 2:**
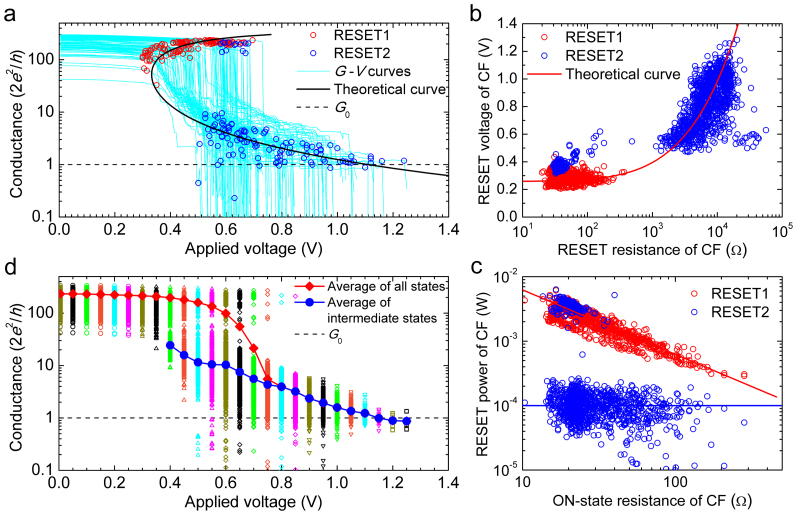
Experimental RESET behavior of a Pt/HfO_2_/Pt RRAM device. (a) 10% *G*–*V* curves randomly selected from 1250 RESET cycles. (b) 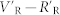 and (c) 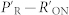 scatter plots for RESET1 (red circles) and RESET2 (blue circles) with data corrected by *R*_S_ = 28 Ω. (d) Evolution of the conductance distribution with *V* in the 1250 successive RESET cycles. The intermediate states are those with conductance below 50*G*_0_.

To reach a holistic understanding of the RESET transient, we need to investigate the details of the RESET1 and RESET2 events and the progressive evolution of the CF conduction properties. Therefore, we have studied the statistics of 1250 successive SET/RESET cycles in a single device. For clarity, only a random selection of 10% of the experimental *G*–*V* curves is reported in Figure 2a, together with the corresponding RESET1 and RESET2 points. Up to the RESET1 point, the conductance is found to decrease continuously due to the increase of the local temperature in the CF^29^,^30^,^33^,^34^,^35^. The conductance of RESET2 point (*G*_R2_) is of the order of a few times the quantum of conductance and reaches the limit of approximate *G*_0_ for those samples that show RESET2 at the higher voltages. In fact, at RESET2, we can represent the CF conductance as *G*_R2_ = *mβG*_0_ where *m* is an integer number representing the number of one-dimensional quantum-mechanical conducting modes and *β* is a coupling parameter (0 < *β* < 1) which can model several effects which limit the conductance of these modes. It has been argued, for example, that each conducting one-dimensional subband under high bias, i.e., in the so-called nonlinear conduction regime, contributes with *βG*_0_ to the CF conductance^40^. In an atomic-scale conducting CF or quantum wire (QW), the voltage mainly drops at the interfaces with the external reservoirs, and the value of *β* is the fraction of voltage that drops at the cathode interface. The value of *β* might change with the actual geometry of the CF and with its coupling to the reservoirs. The presence of impurities in the QW can also reduce the transmission coefficient below unity so that even in the linear regime the contribution to conductance can be smaller than *G*_0_. Experimental results similar to ours, including the observation of integer and non-integer quantized conductance in RRAM, have been recently reported^41^,^42^,^43^,^44^,^45^,^46^. The value of *β* ~ 0.5 can be associated with a rather symmetric voltage drop at both CF/electrode interfaces. Both our work and another recent work^41^ proved that most of the conductance of the intermediate states concentrates around the integer multiples of ~0.5*G*_0_. For this reason, in our simulator, we have also selected 0.5*G*_0_ for each individual *G*_CF_ drop in the RESET process.

Figures 2b and 2c show the CF RESET voltage (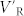) versus the CF RESET resistance (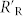) and the CF RESET power (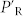) versus the CF ON-state resistance (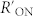) scatter plots, respectively. 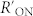 represents the CF resistance at the beginning of the RESET experiment while 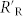 is the CF resistance just at the RESET point. Both resistance values are very similar when considering the RESET1 point, but they can be radically different for RESET2 because the CF has been partially RESET before the RESET2 point. Figure 2b shows that 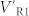 is roughly independent of 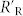 (see S2 of the Supporting Information) while 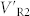 monotonously increases with 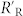. Figure 2c demonstrates the relation 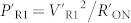 by assuming 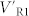 = 0.25 V and a constant 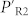 independent of 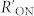. The small number of blue circles that appear on top of the red circles in Figures 2b and 2c correspond to the cycles showing abrupt RESET. Figures 2b and 2c demonstrate that RESET1 is controlled by the voltage applied to the CF while RESET2 is controlled by the power dissipated in the CF. Thus, the RESET transition is divided into two regimes: the voltage controlled RESET1 regime and the power controlled RESET2 regime. The ON-resistance is a very important parameter influencing the statistical variations of the RESET process. Figure S2 in Supplementary Information further shows that both the RESET1 voltage and the current before data correction scale with 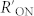
30, and thus it is important to control the variation of ON-resistance to obtain highly uniform distributions of the RESET parameters. Some recent works^22^,^23^ have reported similar scaling behaviors and the scaling theory of the RESET1 voltage and the current in unipolar Pt/NiO/Pt devices. The observed crossover behaviors in the scaling were explained in terms of the connectivity of conducting filaments by analogy with percolation theory. Alternatively, in our work, we explain the two experimental 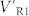 versus 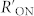 scaling regimes in terms of the thermal dissolution model of RESET^29^,^30^,^35^.

**Figure 3:**
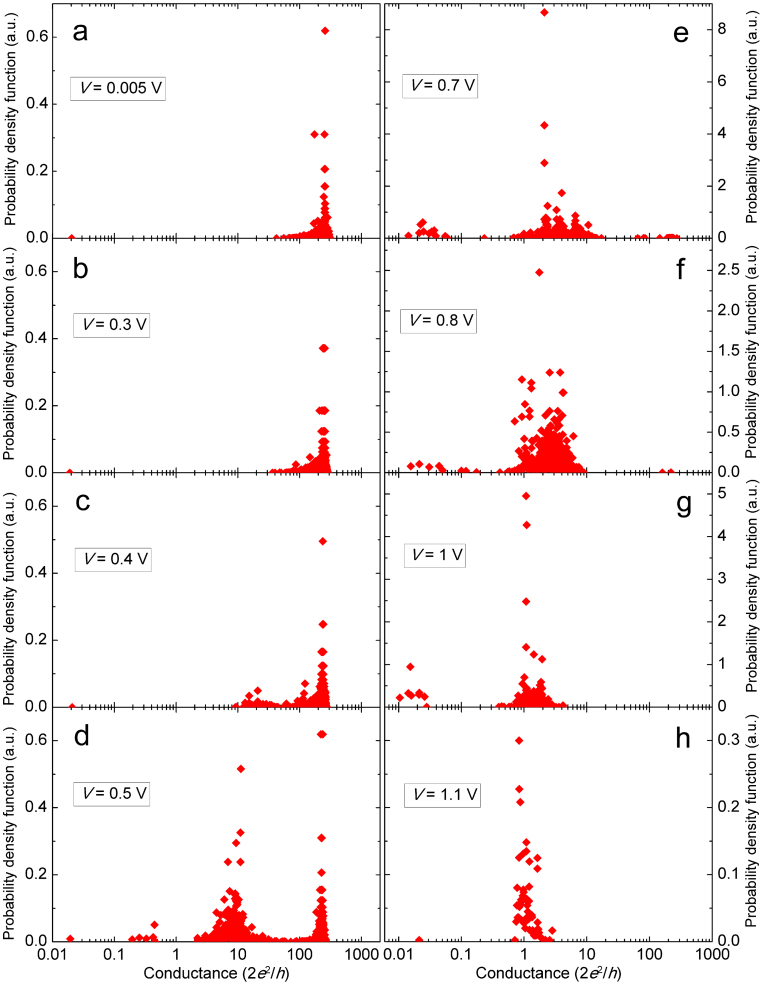
The probability density function of the normalized conductance (*n* = *I*/(*G*_0_*V*)) for some illustrative values of *V*. The probability density function of *n* is expressed as Δ*F*/Δ*n*, where *F* is the cumulative probability of each *n* ordered in increasing order. At each typical voltage, there are one or several characteristic conductance peaks. With the increase of *V*, the characteristic conductance peaks shift towards lower values, finally reaching approximately 1*G*_0_ before the final CF rupture associated with RESET2.

To further explore the RESET transients, we studied the evolution of the statistical distribution of the conductance as a function of the applied voltage (*V*), as shown in Figure 2d. Some voltage snapshots of the conductance distribution are shown in Figure 3. Before *V* = 0.4 V, each cycle almost maintains its initial high conductance state (*G* ~ 300*G*_0_) such that the distribution remains practically unchanged except for a small shift related to a temperature increase. The high initial conductance is kept by a fraction of cycles when *V* reaches 0.5 to 0.7 V and even 0.8 V. The occurrence of some completely abrupt RESET transitions (RESET1 = RESET2) is also appreciated starting at *V* = 0.5 V because some cycles already show *G* < *G*_0_. From *V* = 0.4 V, many cycles suffer an abrupt conductance drop (RESET1), and a peak of intermediate conductances (<50*G*_0_) appears in the distribution. After the initial jump, the RESET is progressive, and the peak of the distribution moves downwards to lower conductance. The average conductance of the intermediate states decreases with *V*. At approximately *V* = 1.1 V, the conductance of all of the remaining cycles is approximately *G*_0_, indicating that the CF behaves as a single-channel quantum wire at the final stage of dissolution.

The experimental observations shown in Figures 1 and 2 can be interpreted well by the thermal dissolution model^29^,^30^,^33^,^34^,^35^. This model assumes that RESET occurs due to the temperature-activated oxidation of the oxygen-deficient CF. The CF temperature is determined by the balance between the energy dissipation in the CF and heat evacuation, which can be phenomenologically modeled through a thermal resistance (*R*_th_). According to Ielmini et al.^35^, the CF RESET voltage can be written as 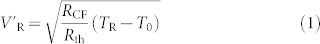
where *R*_CF_ is the CF electrical resistance, *T*_0_ is room temperature, and *T*_R_ is the critical RESET temperature. A value of *T*_R_ = 750 K provides a good fit to the 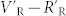 scatter plot in Figure 2b. Eq. (1) also shows that a key element to explain the experimental results is an adequate modeling of *R*_th_, which should include the contributions of two different heat evacuation paths: the parallel thermal resistance (*R*_||_) related to the heat loss along the CF, and the perpendicular thermal resistance (*R*_⊥_) accounting for the heat transfer from the CF surface to the surrounding oxide. Thus, the combined thermal resistance *R*_th_ can be calculated as 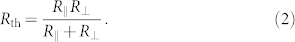
According to the Wiedemann-Franz law^33^, *R*_||_ is proportional to *R*_CF_ and can be written as *R*_||_ = *R*_CF_/(8*LT*_R_), where *L* = 2.45 × 10^−8^ W Ω K^−2^ is the Lorentz number. On the other hand, a constant *R*_⊥_ can be assumed as a first order approximation. Before RESET1 when *R*_CF_ is small, the heat loss in the transport direction is very efficient, and *R*_th_ is mainly determined by *R*_||_, so the ratio *R*_CF_/*R*_th_ remains constant, resulting in a 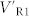 independent of *R*_CF_. On the contrary, once *R*_CF_ reaches sufficiently high values due to the partial CF dissolution, *R*_⊥_ becomes dominant, and *R*_CF_/*R*_th_ increases with *R*_CF_. Thus, according to Eq. (1),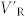 tends to increase with *R*_CF_ in this regime (Figure 2b), while the RESET power 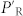 becomes practically independent of *R*_CF_ (Figure 2c). Thus, we can say that the RESET enters into a power-controlled regime. The existence of these two RESET regimes was suggested by a subtle change of 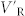 reported in Ref. 35 where only RESET1 was considered, but the initial CF resistance was varied using either a partial RESET method or a current limited SET using a 1T1R configuration. The results shown in Figures 2b and 2c are obtained with a different methodology because we consider the whole RESET transient following the progressive change of *R*_CF_ and including both RESET1 (with a rather narrow distribution of *R*_CF_) and RESET2 (equivalent to the RESET of CFs with much higher and widely-distributed *R*_CF_), instead of varying the initial CF resistance. Our results provide a much clearer picture of the dependence of the RESET parameters on *R*_CF_ and provide stronger experimental support to the CF thermally-assisted dissolution model.

The series resistance, *R*_S_, plays an important role in the occurrence of abrupt or progressive RESET event and the corresponding change of the CF morphology. Considering the series combination of the CF and *R*_S_, the applied voltage at RESET can be calculated as 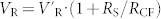, and the total normalized conductance is given by *n* = 1/[(*R*_CF_ + *R*_S_)*G*_0_]. Eliminating *R*_CF_ from these two equations and Eq. (1), we obtain the *V*_R_–*n* relation as 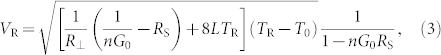
which is compared to experiments in Figure 2a. The calculated 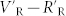 curve is also plotted in the experimental scatter plot of Figure 2b. In both cases, the experiments are nicely fitted by assuming *R*_⊥_ = 5 × 10^6^ K W^−1^, a value which is lower than that estimated for NiO in Ref. 35. The non-zero *R*_S_ explains why the *n*–*V*_R_ curve in Figure 2a has two branches. In the upper branch, a significant fraction of the applied voltage drops on *R*_S_ such that 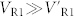. As 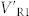 is constant according to Eq. (1), *V*_R1_ increases with *G*_CF_. In the lower branch, the *V*_R_–*G*_CF_ trend is the opposite because *R*_S_ ≪ *R*_CF_ and *R*_th_≈*R*_⊥_. The upper branch explains the first abrupt RESET event and also explains the change of the CF size from inset A to B in Figure 1. The non-zero *R*_S_ plays an important role in the existence of the upper branch and the existence of the RESET1 events. If *R*_S_ = 0, *V*_R1_ will equal to 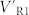, so *V*_R1_ will become constant according to Eq. (1), and thus the upper branch and the RESET1 events will disappear, as shown in Figure S3 in Supplementary Information. The lower branch explains the progressive RESET phase that precedes the final RESET2 drop and the change of the CF size from inset B to C in Figure 1 as well. When the RESET1 point is reached, CFs with high and low conductance display different behaviors. If the initial conductance is relatively low and located in the lower branch, for each small increment of *V*, the conductance shows small drops because any conductance drop leads to an increase of the voltage required to maintain *T*_R_. In other words, there is a negative feedback process that limits the conductance drop at constant voltage, thus leading to the progressive RESET behavior. On the contrary, if the CF conductance is initially high and the RESET occurs in the upper branch, any small drop of the conductance causes an increase of *V*_CF_ because a smaller fraction of the applied voltage drops in the series resistance. The increase of *V*_CF_ produces a subsequent increase of the CF temperature (*T*_CF_), which is maintained above *T*_R_ until the CF conductance reaches the lower branch. Thus, the series resistance effects explain the large RESET currents and the large and abrupt conductance drops observed at RESET1 in those cycles with a high initial CF conductance. The magnitude of the abrupt conductance drop registered at RESET1 is as large as necessary to reach the lower branch of the curve (with a certain statistical dispersion). The magnitude of this abrupt conductance drop is related to *G*_ON_ and it is determined by the voltage-dependent separation between the two conductance branches. Once the conductance has dropped from the high branch to the low branch, the progressive RESET process begins. However, there is also a non-negligible probability of the CF to be completely ruptured at RESET1 due to damage propagation effects. This abrupt RESET is more probable when the initial CF conductance is very high and the RESET current reaches the maximum values. In this case, there is no progressive RESET following the abrupt RESET1 event, which directly decreases the CF conductance well below *G*_0_.

## Discussion

**Figure 4:**
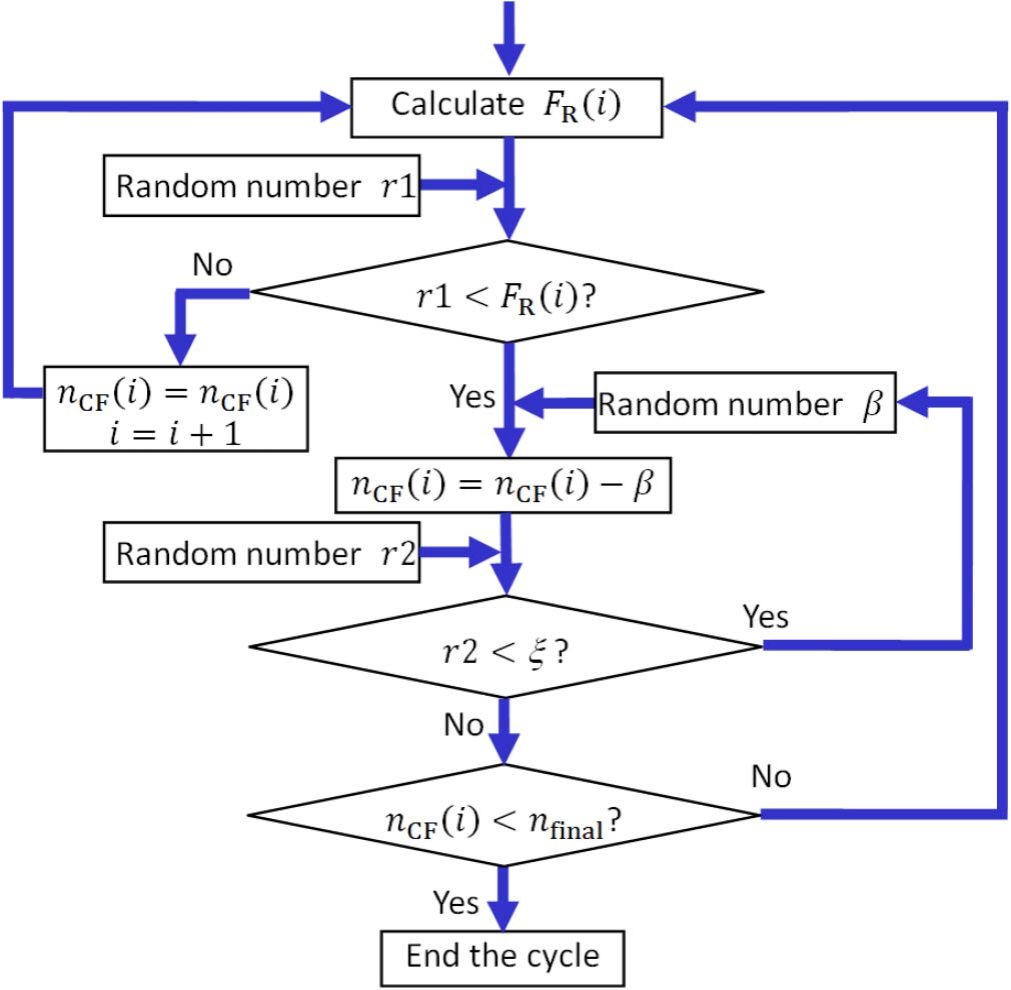
Flowchart of the Monte Carlo simulator used for the simulation of RESET switching cycle. In the Pt/HfO_2_/Pt RRAM device, the RESET probability *F*_R_ is calculated according to the thermal dissolution model. The evolution of the CF conductance is stochastically decided by the comparison between *F*_R_ and the random number *r*1. By introducing a parameter *ξ* to describe the correlation between successive RESET events, large conductance drops are also reproduced. In our simulations (Figures 5 and 6), a value of *ξ* = 0.85 is selected, the conductance drop (*β*) at each time is approximately 0.5, obeying a Gaussian distribution with a mean of 0.5 and a standard deviation of 0.1, and *n*_final_ is approximately 1 obeying a Gaussian distribution with a mean of 1 and a standard deviation of 0.3.

To help understand the experimental RESET statistical results, we developed a simulator based on the thermal dissolution model^29^,^30^,^33^,^34^,^35^. Because the RESET is related to the random occurrence of single-atom events at the nanoscale (i.e., an oxygen atom diffuses and recombines with one vacancy of the CF), the simulation of the RESET process needs to be stochastic. In other words, the Monte Carlo method is necessary in our simulation. Figure 4 shows the flowchart of our proposed Monte Carlo simulator. We simulate the application of a staircase voltage ramp, as used in the actual experiments. In each time interval, the applied voltage is kept constant as *V*(*i*) = *I* × Δ*V*, and the evolution of the normalized CF conductance (*n*_CF_) is stochastically decided by generating random numbers and comparing them with the RESET probability, *F*_R_(*i*). In the thermal dissolution model, the RESET is considered to occur by the out-diffusion of the conducting defects (i.e., oxygen vacancies) when the local CF temperature *T*_CF_(*i*) is close enough to a critical value, which we call the RESET temperature *T*_R_. In this situation, the average number of RESET events occurring during a fix time interval (such as the *i*^th^ interval of our MC simulation) is also thermally activated and can be expressed as 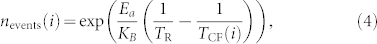
where *E*_a_ is the activation energy (likely related to the process of oxygen diffusion), *K*_B_ is the Boltzmann constant and *T*_CF_(*i*) is the temperature of the CF during the *i*^th^ interval. Considering that the RESET events occur at random, the probability that at least one RESET event occurs during the *i*^th^ simulation interval, *F*_R_(*i*), can be calculated according to the Poisson distribution: 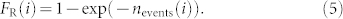
According to this probability distribution, ~63% of the samples have suffered RESET when the CF temperature reaches *T*_R_. The width of the distribution of temperature at RESET depends on the activation energy. The higher is *E*_a_, the narrower the distribution of RESET temperature. In the simulation, the CF temperature *T*_CF_(*i*) is related to the CF voltage according to 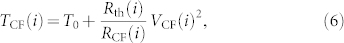
where *V*_CF_(*i*) is calculated as 
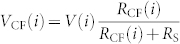
. Before the RESET1 point, *R*_CF_ is assumed to change with temperature as *R*_CF_(*i*) = *R*_0_[1 + *γα*(*T*_CF_(*i*) − *T*_0_)], which is typical for metals and degenerately doped semiconductors^35^. Here, *γ* is the geometrical coefficient corresponding to the shape of the CF, and *α* is the resistance-temperature coefficient. The fitting of experiments yields *γα* ~ 6 × 10^−4^ K^−1^.

In each time interval, the occurrence of a RESET event is decided by comparing a random number with *F*_R_(*i*). After one of these partial RESET events, *G*_CF_ drops by 0.5*G*_0_ (i.e., *n*_CF_ drops by 0.5) on average^39^,^40^,^41^. Larger conductance drops are also considered by introducing correlations between successive events. These discrete conductance drops are consistent with the idea that the CF consists of a percolating web of even smaller atomic-size filaments formed by oxygen vacancy chains with a conductance of the order of *G*_0_^38^. Once *G*_CF_ is changed, *T*_CF_ and *F*_R_ are recalculated, and the process is repeated as many times as dictated by the comparison of successive random numbers (*r*1) with the evolving *F*_R_(*i*), until *G*_CF_ drops below *G*_0_, which is considered to correspond to the complete CF rupture.

**Figure 5:**
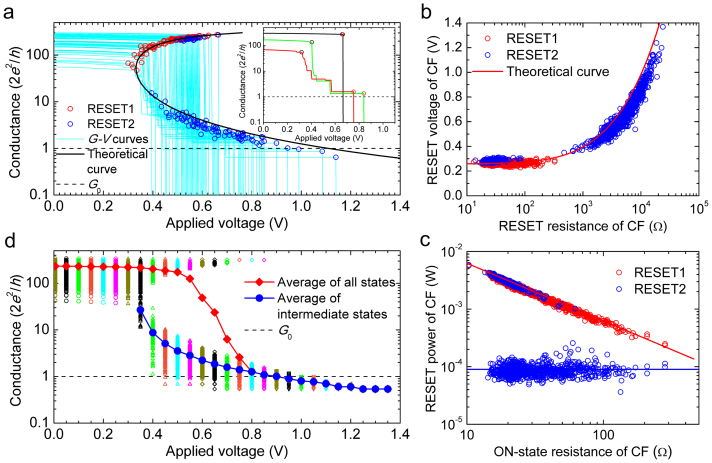
Simulation results of the RESET switching with fixed *E*_a_ and *R*_⊥_. (a) 10% *G*–*V* curves randomly selected from 1250 RESET cycles. The inset shows three typical *G*–*V* curves similar to those in Figure 1. (b) 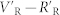 and (c) 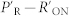 scatter plots for RESET1 (red circles) and RESET2 (blue circles). (d) Evolution of the conductance distribution with *V* in the 1250 successive RESET cycles. In this simulation, *E*_a_ = 1 eV, *R*_⊥_ = 5 × 10^6^ K W^−1^.

The results are found to be most sensitive to the values of *R*_⊥_ and *E*_a_. Figure 5 shows the simulation results with fixed values for all of the model parameters, in particular, *T*_R_ = 750 K, *R*_⊥_ = 5 × 10^6^ K W^−1^ and *E*_a_ = 1 eV, which is a typical value in oxide-based RRAM devices^33^. The same set of initial CF conductance values as those found in the 1250-cycle experiment was used as the starting value of the normalized CF conductance for the simulation. The comparison of Figures 5 and 2 reveals that the complete phenomenology of RESET is nicely captured by the stochastic simulation, including the initial abrupt event after RESET1, the subsequent progressive RESET phase (Figure 5a), and the overall evolution of the conductance distribution (Figure 5d). Moreover, the simulation reproduces the resistance-independent 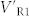 (Figure 5b) that defines the voltage-controlled RESET regime, and the constant 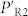 (Figure 5c) contributing to the power-controlled regime. Even the random occurrence of fully progressive and completely abrupt RESET transients, corresponding to the lowest and highest initial CF conductance, respectively, is reproduced (inset in Figure 5a). These results demonstrate that the thermal dissolution model provides a solid framework not only for the initial phase of the RESET process (RESET1), as considered in previous works^33^,^34^,^35^, but also to explain the main features of the whole RESET transient, including the abrupt and progressive RESET phases.

**Figure 6:**
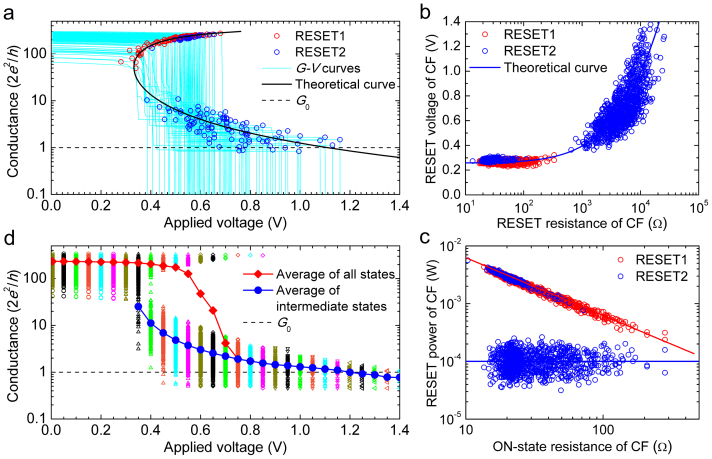
Simulation results of the RESET switching with variable *E*_a_ and *R*_⊥_. (a) 10% *G*–*V* curves randomly selected from 1250 RESET cycles. (b) 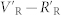 and (c) 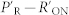 scatter plots for RESET1 (red circles) and RESET2 (blue circles). (d) Evolution of the conductance distribution with *V* in 1250 RESET cycles. In this simulation, *E*_a_ is assumed to obey a constant distribution between 0.8 eV and 1.4 eV, and *R*_⊥_ is assumed to range between 2 × 10^6^ K W^−1^ and 1 × 10^7^ K W^−1^ and obey a Gaussian distribution with a mean of 4 × 10^6^ K W^−1^ and a standard deviation of 3 × 10^6^ K W^−1^.

The statistical dispersion of the simulated RESET parameters in Figure 5 is significantly smaller than that found in the experiments (Figure 2). Thus, random variations of *R*_⊥_ and *E*_a_ have been incorporated into the MC code, which is reasonable because the CF shape is different in each cycle and the local atomic-scale variations of the CF configuration can induce strong changes in the local transport and chemical properties. The simulation results in Figure 6 are much more similar to the experimental results.

In summary, the voltage-ramp induced RESET of HfO_2_-based RRAM devices is reported to show both abrupt and progressive phases preceding the final complete CF rupture. Different RESET phenomenology has been reported as a function of the initial CF conductance. High conductance paths usually show a first voltage-controlled abrupt RESET jump followed by a power-controlled progressive RESET phase. The most resistive CFs directly enter into the progressive RESET phase. These two phases are related to two different thermal evacuation regimes in which the heat flows through the CF in the electron transport direction and from the CF surface to the surrounding oxide, respectively. Based on the thermally activated CF dissolution model, we constructed a Monte Carlo simulator that remarkably reproduces the main experimental features of the RESET process, including the abrupt and progressive phases and the evolution of the conductance distributions. The results strongly support the thermal dissolution model, improve our understanding of the progressive rupture of the CF under unipolar switching conditions and highlight the impact of the series resistance on the RESET dynamics. The progressive RESET process increases the stress requirements for the full transition to the HRS, which is not advantageous to uniformity of the device. This issue needs to be accounted in the design of the RESET operation algorithms to avoid RESET failures in the one-bit/cell device. Current sweeping mode can facilitate effectively eliminating the intermediate states in RESET transition. In the practical application, the RRAM device is operated in pulse mode and the probability of the progressive RESET will be strongly suppressed at the high stress voltages required for fast pulse RRAM operation. On the other hand, filament engineering such as introducing metal NC or other electric-field-concentrating initiators to control the CF formation path is advantageous to improve the uniformity and reliability of RRAM. Our methodology reported in this work can be easily extended to the RESET and SET switching in all kinds of RRAM devices, which will guide people to understand the physics of RS behavior more clearly and optimize the performances of RRAM device with effective methods.

## Methods

### Device fabrication

The studied RRAM device with a Pt/HfO_2_/Pt structure was fabricated onto a tungsten plug. The 10-nm-thick HfO_2_ resistive switching layer was deposited by atomic layer deposition (ALD) at 350°C on the Pt bottom electrode (BE), followed by Pt top electrode (TE) deposition and patterning by photolithography and etching. Pt BE and TE were deposited by physical vapor deposition (PVD).

### Measurements

To initiate the resistive switching, a preliminary electroforming process similar to a soft dielectric breakdown event is required. It is a one-time writing process at voltage higher than regular operation voltage. After the electroforming operation, long lasting repetitive cycling experiments were performed using voltage ramp stress (VRS) both for SET and RESET with an Agilent 4155C semiconductor parameter analyzer. The *I*–*V* curves in the 1250 successive SET/RESET cycles were recorded to a device with an area of 1 µm^2^. During the SET transition, a compliance current with 1 mA was applied by 4155C to avoid the hard breakdown of the HfO_2_ layer. RESET test was stopped when the HRS resistance was 300 or 350 times higher than the LRS resistance.

## Author Contributions

S.L., E.M. and J.S. did the data analysis and interpreted the results. C.C., L.P. and J.B. fabricated the devices and performed the voltage-ramp cycling experiments. S.L. and X.L. performed the Monte Carlo simulations. J.S., S.L., F.P. and M.L. co-wrote the manuscript. All authors critically read and contributed to the manuscript preparation. J.S. and M.L. coordinated and supervised the whole work.

## Supplementary Material

Supplementary Information Supplementary Information
