# Supplementary material for: Voltage and Power-Controlled Regimes in the Progressive Unipolar RESET Transition of HfO2-Based RRAM
Source: Sci Rep. 2013 Oct 14;3:2929. doi: 10.1038/srep02929 (PMC3796310; doi:10.1038/srep02929)
Supplement: Supplementary Information [file srep02929-s1.pdf]

Supplementary Information

Voltage and Power-Controlled Regimes in the Progressive Unipolar RESET Transition of HfO_2-Based RRAM

Shibing Long[1,2], Luca Perniola[3], Carlo Cagli[3], Julien Buckley[3], Xiaojuan Lian[2], Enrique Miranda[2], Feng Pan[4], Ming Liu[1 *] & Jordi Suñé[2 *]

[1]*Lab of Nanofabrication and Novel Device Integration, Institute of Microelectronics, Chinese Academy of Sciences, Beijing 100029, People's Republic of China*

[2]*Departament d'Enginyeria Electrònica, Universitat Autònoma de Barcelona, Bellaterra 08193, Spain*

[3]*CEA, LETI, MINATEC Campus, 17 rue des Martyrs, F-38054 Grenoble Cedex 9, France*

[4]*Laboratory of Advanced Materials, Department of Materials Science and Engineering, Tsinghua University, Beijing 100084, People's Republic of China.*

**To whom correspondence should be addressed. E-mails:* liuming@ime.ac.cn or jordi.sune@uab.es

1. Estimation of the series resistance.

The series access resistance of the studied Pt/HfO_2/Pt RRAM device consists of two parts. One is the parasitic resistance associated with cables and contacts of the experimental setup, which is approximately 18 Ω. The other is the spreading resistance (Maxwell resistance) related to the funneling of the current lines from the Pt metal contacts to the nanometer wide CF, which has been estimated to be approximately 10 Ω. The total series resistance (R_S) is approximately 28 Ω. **Figure S1** shows the calculated Maxwell resistance (R_M) as a function of the CF radius. The inset is a schematic structure of the device.

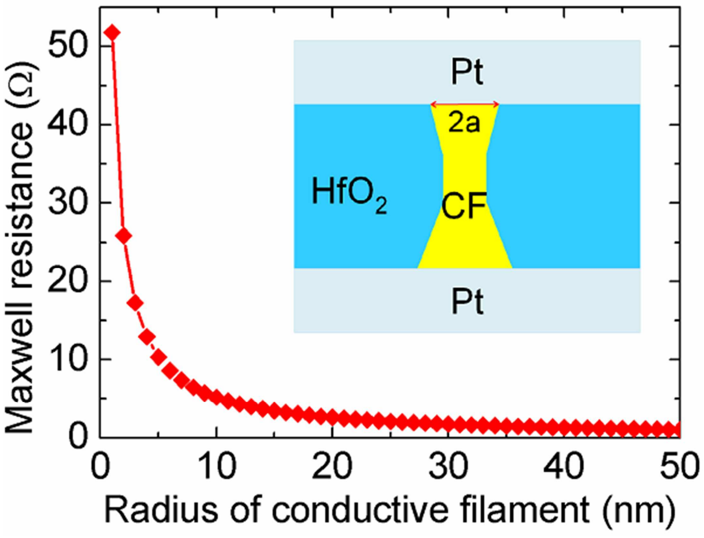

Figure S1. The calculated Maxwell resistance of the contact interface between the narrower end of CF and Pt electrode in Pt/HfO_2/Pt RRAM device through the equation: $R_M = \frac{\rho}{2a} = \frac{1}{2\sigma a}$, where a is the radius of the narrower end of CF and ρ and σ are the resistivity and conductivity of Pt bulk materials, respectively. The inset is the schematic structure of the device with a CF formed in the ON-state. From the CHEMIX Periodic Table, the conductivity of Pt bulk materials is 9.96×10^6 $ohm^{-1}m^{-1}$. The diameter of the CF (originated from oxygen vacancies) directly observed by TEM in Ref. S1 is approximately 5 nm. If we choose a = 5 nm, then R_M = 10 Ω.

2. The statistics of RESET1 voltage and current before and after data correction by the series resistance.

When the data are not corrected by R_S, V_{R1} decreases with R_{ON}. After data correction by R_S = 28 Ω, the RESET1 voltage dropped on the CF, V'_{R1}, becomes constant with the CF resistance in the ON-state, R'_{ON} [S2]. This constant V'_{R1} behavior is physically significant and can be explained in terms of the thermal dissolution model of RESET[S3]. In this model, RESET is considered to occur by the out-diffusion of the conducting defects (i.e., oxygen vacancies) when the local CF temperature reaches a critical value T_R. Taking into account the balance

between Joule dissipation and heat evacuation, the basic equation of the model is $T_{\mathrm{R}} = T_0 + (R_{\mathrm{th}}/R'_{\mathrm{ON}}){V'_{\mathrm{R1}}}^2$, where V'_{R1} is the RESET1 voltage dropped in CF, T_0 is the operation temperature and R_{th} is the thermal resistance describing heat dissipation from the CF to the environment. If R'_{ON} is low enough, $R_{\mathrm{th}} \propto R'_{\mathrm{ON}}$ due to the Wiedemann–Franz law, and V'_{R1} is predicted to be independent of R'_{ON}, as shown by the red circles in Figure S2(a). In consequence, in the thermal dissolution model, the thermal dissolution process of CF is voltage controlled, i.e. the voltage determines the local temperature and hence the rate of diffusion of oxygen species. Before data correction, the RESET1 current decreases with R_{ON}, with the slope gradually decreasing. After data correction, I_{R1} decreases linearly with R'_{ON}[S2] due to the constant V'_{R1} and due to the fact that $I_{\mathrm{R1}} \approx V'_{\mathrm{R1}}/R'_{\mathrm{ON}}$ because the $I - V$ curves are nearly linear before reaching the RESET1 point[S2].

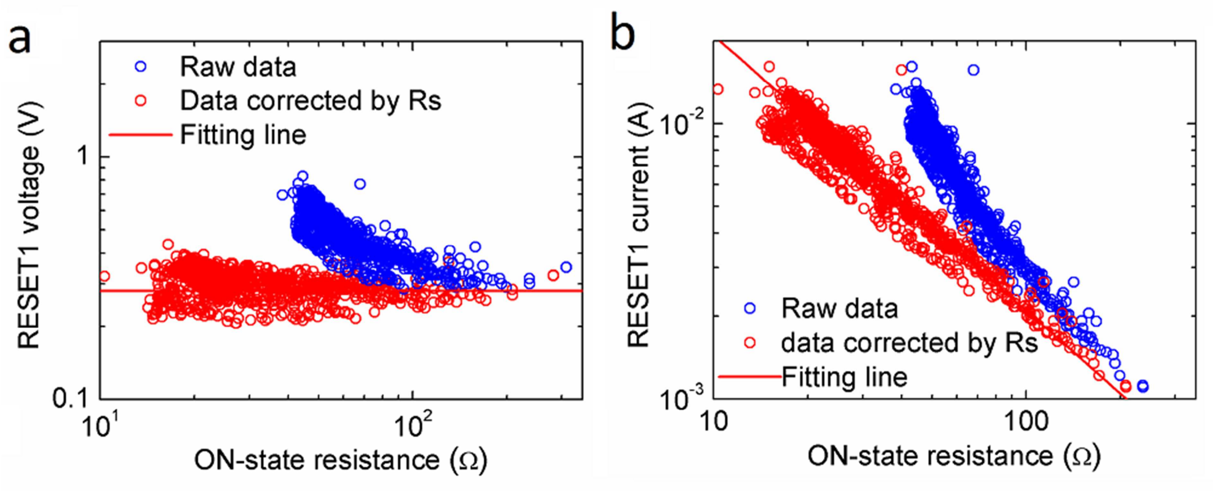

Figure S2. The ON-resistance dependences of RESET1 voltage (a) and RESET1 current (b) before and after data correction by $R_{\mathrm{S}} = 28\ \Omega$. The relation between the corrected and uncorrected parameters is $R'_{\mathrm{ON}} = R_{\mathrm{ON}} - R_{\mathrm{S}}$ and $V'_{\mathrm{R1}} = V_{\mathrm{R1}} - I_{\mathrm{R1}}R_{\mathrm{S}}$ according to the series connection of CF and series resistance.

3. The role of R_S in determining the branch structures of the theoretical $V_R - n$ curves.

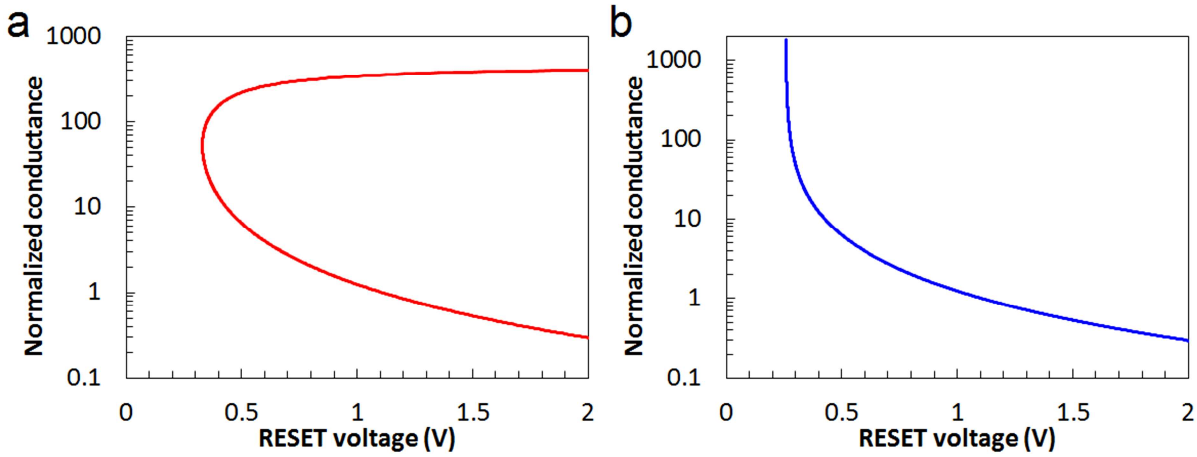

Figure S3. Theoretical $V_R - n$ curves plotted from Eq. (3) in the manuscript when (a) R_S = 28 Ω and (b) R_S = 0. The same values of the parameters as those in the manuscript are used. In (a), the $V_R - n$ curve has the upper and lower branch because the the non-zero R_S plays an important role. In (b), as R_S = 0, the upper branch disappers.

References

S1. Kwon, D. H. *et al*. Atomic structure of conducting nanofilaments in TiO_2 resistive switching memory. *Nature Nanotech.* **5**, 148−153 (2010).

S2. Long, S. *et al*. Cycle-to-cycle intrinsic RESET statistics in HfO_2-based unipolar RRAM devices. *IEEE Electron Device Lett.* **34**, 623−625 (2013).

S3. Ielmini, D. *et al*. Physical models of size-dependent nanofilament formation and rupture in NiO resistive switching memories. *Nanotechnology* **22**, 254022 (2011).
